# Presurgical cognitive status in patients with low‐grade glioma and epilepsy: Testing the effects of seizures, antiseizure medications, and tumor localization

**DOI:** 10.1002/brb3.2560

**Published:** 2022-04-04

**Authors:** Ilaria Guarracino, Giada Pauletto, Tamara Ius, Francesca Palese, Miran Skrap, Barbara Tomasino

**Affiliations:** ^1^ Polo FVG, San Vito al Tagliamento, PN Scientific Institute IRCCS “Eugenio Medea,” Italy; ^2^ Unità Operativa di Neurologia Azienda Sanitaria Universitaria del Friuli Centrale Udine Italy; ^3^ Unità Operativa di Neurochirurgia Azienda Sanitaria Universitaria del Friuli Centrale Udine Italy; ^4^ Servizio di Igiene e Sanità Pubblica Dipartimento di Prevenzione San Daniele del Friuli Azienda Sanitaria Universitaria del Friuli Centrale Udine Italy

**Keywords:** epilepsy, glioma, neuropsychology, tumor‐related epilepsy, voxel‐based lesion‐symptom mapping

## Abstract

**Background:**

Low‐grade gliomas (LGGs) are frequently associated with epilepsy. There are few studies addressing the impact of seizures, antiseizure medications (ASMs), and lesion localization on presurgery cognitive functioning.

**Methods:**

We tested the relation between the above‐mentioned variables in a continuous series of 73 young patients (mean age 38.3 years ± 11.7) affected by LGGs and epilepsy. The anatomical areas, involved in this sample, were the left insula with surrounding cortical and subcortical areas, the right precentral gyrus/rolandic operculum, and the white matter and cortical regions beneath.

**Results:**

Patients’ presurgery cognitive status was within the normal range, with borderline performance for some tasks. We tested whether lower scores were related with lesion or with epilepsy‐related factors. Multiple regression identified variables that predict test scores. The Token test score was predicted by a model (*p* = .0078) containing the DT2T1 MRI, corrected for seizure features. Object naming performance was predicted by a model (*p* = .0113) containing the localization, the DT2T1 MRI, corrected for sex, EEG, and onset. Verbal fluency score was predicted by a model (*p* = .0056) containing the localization and the DT2T1 MRI, corrected for AEDs and EEG. Working memory score was predicted by a model (*p* = .0117) containing Engel class, the DT2T1 MRI, corrected for sex. Clock drawing score was predicted by a model (*p* < .0001) containing the Engel class, AEDs, and EEG. TMT A score was predicted by a model (*p* = .0022) containing localization, corrected for EEG. TMT B‐A score was predicted by a model (*p* = .0373) containing localization. Voxel Lesion Symptom Mapping analyses carried out on patients’ lesion volumes confirmed that patients’ level of performance correlated with lesion‐related variables.

**Conclusion:**

This preliminary study indicates that the presurgical level of performance for language tasks and for cognitive flexibility and shifting is mainly predicted by lesion‐related variables, working memory by both lesion and epilepsy‐related variables. Epilepsy clinical and instrumental characteristics predicted performance for visuospatial planning.

## Graphical abstract

We tested the effects of seizures, antiseizure medications (ASMs) and lesion localization on presurgery cognitive functioning in a continuous series of 73 young patients affected by LGGs and epilepsy. This preliminary study indicates that the presurgical level of performance is related to lesion‐related variables such as tumor localization. Epilepsy clinical and instrumental characteristics did not significantly correlate with any of the cognitive outcomes

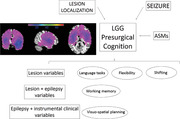

## 1 INTRODUCTION

There are consolidated data showing that neuropsychological alteration is a well‐recognized complication of epilepsy (Lin et al., 2012). It has also been argued that, since epilepsy involves disruption of large‐scale networks, it may alter the interactions between the cognitive domains which are supported by those networks (Kellermann et al., 2016), leading to several cognitive alterations, such as memory, language, praxis, executive functions, and social cognition. Cognitive impairments differ according to the site of epileptic focus, the duration and the type of epilepsy. Cognitive alterations in tumor‐related epilepsy (TRE) have not been widely investigated, especially in the sub‐group represented by low‐grade glioma (LGG)‐related epilepsy. There is one study (Tucha et al., 2000) in which authors tested 139 patients with brain tumors of the frontal or temporal lobes, immediately after diagnosis and reported impairments of executive functions in 78% of patients, memory and attention deficits were found in 60% of patients. Interestingly, authors reported that there was no effect of anticonvulsant drugs on cognition. The relationship between LGG and presurgical neuropsychological changes is established in the literature (Antonsson et al., 2018; Antonsson et al., 2018; Racine et al., 2015; Teixidor et al., 2007; Tomasino et al., 2018; van Kessel et al., 2017). There is, however, an issue in interpreting the neurocognitive functioning of patients with LGG prior to surgical treatment, namely, the possible effect of epilepsy and antiseizure medications (ASMs) on patients’ neuropsychological status. In the majority of cases (otherwise, glioma are defined incidental nonepileptic), LGG patients are affected by TRE. For instance, in a retrospective observational study with 1509 patients, the authors (Pallud et al., 2014) reported that 89.9% of them had experienced seizures at the time of tumor diagnosis.

The way TRE could affect the neurocognitive status is twofold: a direct influence, when seizures arise from the tumoral and peritumoral areas, potentially influencing the functions supported by the region. There is a relationship between seizure incidence and LGG localization. The incidence is higher in cortical regions (56%) than in subcortical regions (15%) (Rudà et al., 2010). An indirect influence of epilepsy is expressed by functional reorganization of areas affected by seizures (Elger et al., 2004). The third way of TRE affecting the presurgical neurocognitive status is via ASMs. Since the presence of seizures affects significantly patients’ quality of life, it is mandatory to reduce seizures frequency and severity and to obtain, when possible, seizure‐freedom (Maschio, 2012). Indeed, inadequate or postponed epilepsy treatments can increase cognitive deterioration (Jokeit & Ebner, 1999; Maschio, 2012). ASMs could, in turn, affect the neurocognitive functioning. Often, ASMs side effects negatively affect the perception and the quality of life of patients (Maschio, 2012; Rahman et al., 2015). Among the most used drugs in TRE, there are levetiracetam, lacosamide, valproic acid, and perampanel. Mono‐ or polytherapy may also differentially affect patients’ neuropsychological status. In the literature, however, there are contradictory evidence for an association between drug treatment and possible effects on cognitive functioning.

In the present study, we aim to evaluate the presurgical neuropsychological status of patient with TRE, and the role of localization, volume, seizure features, and drug treatment on their cognitive functioning. Our hypothesis is mainly based on the clinical observation that LGG patients result, during presurgery assessment, generally quite preserved from a cognitive perspective; thus we suggest that features related to seizures and ASMs may have no or just little effect on the cognitive state of LGG patients, affected by TRE.

## 2 METHODS AND MATERIALS

### 2.1 Participants

In the present retrospective study, we included patients with LGG and TRE who have been operated in our department from November 2007 to May 2018.

Inclusion criteria were the following: age >18 years; preoperative magnetic resonance imaging (MRI) suggestive of supratentorial LGG; preoperative neuropsychological assessment; no previous surgery, chemo‐ or radiotherapy; objective evaluation of extent of resection (EOR) preoperatively and postoperatively on MRI images based on T2‐weighted MRI sequences; and diagnosis of TRE.

Exclusion criteria were precedent biopsy and precedent surgery for brain glioma. The local Ethics Committee, Comitato Etico Unico Regionale del Friuli Venezia Giulia, approved this investigation (protocol N.0036567/P/GEN/EGAS, ID study 2540). Considering that the study was retrospective, written consent to participate in the study was not applicable. Written informed consent was obtained for surgery.

All patients underwent presurgical brain MRI, electroencephalography (EEG), and neurological assessment. They were assessed preoperatively with a neuropsychological test battery. The selected cognitive tests were related to the tumor site.

Data collected were demographic, years of education, tumor side and localization, seizure semiology and frequency, number and type of ASMs, preoperative EEG features, seizure outcome 1 year after surgery, tumor volume on T2‐weighted images, and histology.

### 2.2 Neurological assessment and EEG

All patients underwent neurological interview and assessment before surgery, focusing on epilepsy clinical features and treatment. Seizures were classified according to the 2017 International League Against Epilepsy (ILAE) classification (Fisher et al., 2017). For statistical analysis, seizures were dichotomized in focal versus focal‐to‐bilateral seizures.

Preoperative EEG recordings (32‐channel EB Neuro Mizar Sirius system with Galileo NT software, EB Neuro) were performed according to the 10–20 International System, within 7 days before surgery.

EEGs were scored as follows:

– Normal (N): background activity with alpha or faster rhythms, no focal or diffuse slowing, no epileptic discharges.
– Slow (S): alpha or faster rhythms as background with focal or multifocal slow activity, or alpha rhythm mingled with diffuse theta‐delta activity. Epileptic activity was absent.
– Epileptic (E): alpha activity in the background with faster rhythms or mixed with slower activity. Localized or diffused interictal epileptiform abnormalities (spikes, polyspikes, spike‐and‐wave, polyspike‐and‐wave complexes) were present.

### 2.3 MRI structural data

Data were obtained by retrospectively analyzing structural images routinely acquired presurgery. A 3‐T Philips Achieva whole‐body scanner was used to acquire structural data using a SENSE‐Head‐8 channel head coil. Volumes of interest (VOIs) of the patients’ lesions were drawn on their T1 MRI scans using MRIcron software (https://www.nitrc.org/projects/mricron). We then normalized the VOIs to the Montreal Neurological Institute (MNI) space using the “Clinical Toolbox” (https://www.nitrc.org/projects/clinicaltbx/) for SPM8 (https://www.fil.ion.ucl.ac.uk/spm/).

### 2.4 Neuropsychological evaluation

Patients completed neuropsychological testing prior to surgery. Tests appropriate for the left and the right hemisphere LGG were administered to the two groups of patients, with some test presented to both groups. The neuropsychological tests for left hemisphere LGG included Raven Matrices (Basso et al., 1987), objects and verbs naming, word and pseudoword repetition and reading, lexical decision, naming and verb comprehension, phonological discrimination (Battery for the analysis of language disorders; Miceli et al., 1994), word and pseudoword writing (Luzzatti et al., 1994), oral apraxia (De Renzi et al., 1966), ideomotor apraxia (De Renzi et al., 1980), Token test (De Renzi and Faglioni, 1978), digit span forward and backward (Monaco et al., 2015), trail making test (TMT) (Giovagnoli et al., 1996), verbal fluency (Novelli et al., 1996), semantic fluency (Novelli et al., 1996), digit symbol substitution test (Orsini & Laicardi, 1997), and pyramids and palm trees test (Gamboz et al., 2009). The neuropsychological tests for right hemisphere LGG included Raven Matrices (Basso et al., 1987), clock drawing test (Mondini et al., 2011), constructive apraxia (Spinnler & Tognoni, 1987), corsi forward and backward (Monaco et al., 2015), digit symbol substitution test (Orsini & Laicardi, 1997), letter cancellation, star cancellation, barrage, line bisection (behavioral inattention test [BIT; Wilson et al., 1987]), trail making test A‐B (Giovagnoli et al., 1996), little man (Ratcliff, 1979).

### 2.5 Statistical methods

For each cognitive test, we converted the Raw Score (PG) into Correct Score (PC) for age, schooling, and gender. Then each PC was converted in the correspondent Equivalent Score (PE), with a PE = 0 meaning a pathological performance. A score below or equal/above the external nonparametric tolerance limit of adjusted scores corresponds to 0 or to 4 respectively; 1, 2, and 3 are intermediate.

For some cognitive test, the normative study includes only a cut‐off score. In this case, a score under the cut‐off means a performance below the normal range.

Analyses were performed by using SAS software, version 9.4 (SAS, Cary, NC, USA).

Regarding clinical parameters, lesion localization was dichotomized as 0 (pre‐/postcentral area) and 1 (temporo/insular area), the delta calculated on T1 versus T2 MRI images was dichotomized as 0 (<18 mm) and 1 (>18 mm). Similarly, EEG pattern was classified as 0 (normal), and 1 (epileptic), AEDs was dichotomized as 0 (mototheraphy) and 1 (polytheraphy), seizure feat was dichotomized as 1 (nonmotor) and 2 (motor), and postoperative seizure outcome was classified as 0 (Engel Class Ia), and 1 (Engel Classes Ib‐IV).

We first performed a Wilcoxon–Mann–Whitney test, to test whether the medians of the neuropsychological scores (for all the tasks) differed significantly for the analyzed variables. We then performed a bivariate Spearman's correlation analysis between the epilepsy‐related variables. Lastly, predictors strongly associated with test scores in univariate models and variables considered as relevant were included a multivariate logistic regression model, where the backward stepwise entry method was applied. Predictors that were significant at the .05 level were retained in the final model.

#### 2.5.1 Voxel‐based lesion‐symptom mapping (VLSM) analysis

The VOI and behavioral data were analyzed in a voxel‐based lesion‐symptom mapping (VLSM) procedure https://www.nitrc.org/projects/mricron. We used the NPM (nonparametric mapping) software. We set the Brunner Munzel (BM) test, 1000 permutations, only testing voxels damaged in at least 10% individual for each test (10% of patients within that hemisphere for test performed to the LH or RH patients [see Table 2] or 10% of all patients for working memory and short‐term memory). Each patient's accuracy score on the cognitive task was used as continuous behavioral variable. The critical *z*‐value considered as our BM map was *p* < .05 value. Any value in the power map and the BM map exceeding this critical *z*‐value was considered significant.

## 3 RESULTS

### 3.1 Study population

A consecutive series of 73 low‐grade glioma (LGG) patients (48 male, 25 female, mean age 38.3 years ± 11.7; mean education was 13 ± 3.8) entered in the study. All patients had TRE and were pharmacologically treated. Mean presurgical duration of epilepsy was 5.8 ± 5 months. Table 1 shows information about lesion localization, size, onset of the disease, and Table 2 shows patients’ seizure frequency and pharmacological therapy.

**TABLE 1:** Patients’ clinical details

	RH patients (*n* = 42)	LH patients (*N* = 31)
**Gender**	27 M;	22 M;
	15 F	9 F
**Age (mean and SD)**	38.42 ± 11.84 years	38.25 ± 11.46 years
**Education (mean and SD)**	13 ± 3.8 years	
**Localization**	16 Precentral 1 Postcentral 9 Insula 16 Temporal	12 Precentral 4 Postcentral 8 Insula 7 Temporal
**Volume on T2‐weighted images (mean and SD)**	43.88 ± 26.57 mm^3^	45.83 ± 27.87 mm^3^
**Histology**	10 Oligodendroglioma, IDH mutant and 1p/19q codelated	13 Oligodendroglioma, IDH mutant and 1p/19q codelated
	4 Diffuse astrocytoma, IDH wild‐type	3 Diffuse astrocytoma, IDH wild‐type
	28 Diffuse astrocytoma, IDH mutant	15 Diffuse astrocytoma, IDH mutant

**TABLE 2:** Patients’ epilepsy features

	RH patients (*n* = 42)	LH patients (*N* = 31)
**Engel Class 1 year**	31 E.C. I	26 E.C. I
	6 E.C. II	4 E.C. II
	3 E.C. III	1 E.C. III
	2 E.C. IV	0 E.C. IV
**ASMs**	32 Levetiracetam	22 Levetiracetam
	1 Carbamazepine	3 Carbamazepine
	2 Oxcarbazepine	2 Oxcarbazepine
	3 Valproic acid	0 Valproic acid
	4 combination	3 combination
	0 zonisamide	1 zonisamide
**Onset**	24 focal to bilateral	20 focal to bilateral
	17 focal	11 focal
**Seizure features**	3 motor	3 motor
	5 nonmotor sensory	4 nonmotor sensory
	8 nonmotor cognitive	4 nonmotor cognitive
	3 nonmotor_autonomic	0 nonmotor_autonomic
	23 motor_t‐c	20 motor_t‐c
**Frequency**	25 monthly	22 monthly
	15 weekly	7 weekly
	2 daily	2 daily

### 3.2 MRI structural results

**FIGURE 1:**
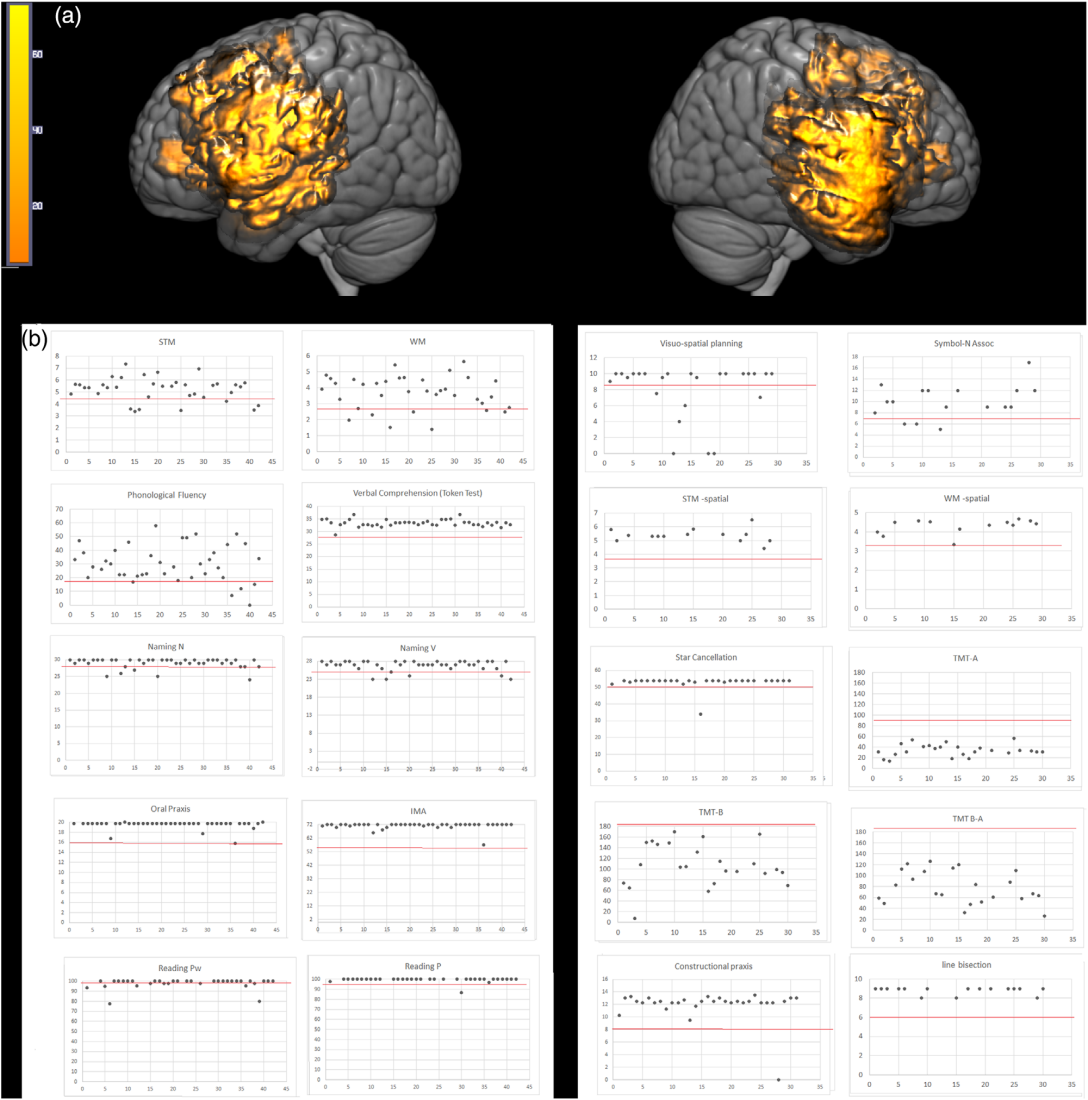
(a) Patients’ lesion VOIs overlay. (b) Patients’ performance at the different tasks divided for the left (left panel) and the right (right panel) hemisphere

The lesion overlay showed that the maximum overlap occurred in the left insula and surrounding cortical and subcortical area, and the right precentral gyrus/rolandic operculum and the white matter and cortical region beneath (see Supplementary Table S1 and Figure 1).

### 3.3 Neuropsychological results

In general, the patients’ presurgery neuropsychological status was within the normal range: at Raven matrices, short‐term memory and working memory, the whole group of patients succeeded. Tasks administered to the RH and the LH groups showed that, for the RH group, all the patients were normal in almost all the measured domains (see Table 3) except for a low number (3/17) of pathological patients at processing speed.

**TABLE 3:** Patients’ cognitive profile

Hemisphere	Task	Total no. of impaired patients	No. of patients with ES = 1 (borderline performance)
RH and LH	Raven matrices	0	2
RH and LH	Short‐term memory	Verbal 7, spatial 0	Verbal 1, spatial 0
RH and LH	Working memory	Verbal 7, spatial 0	Verbal 5, spatial 1
RH	Constructional praxis	1	0
RH	Clock drawing test	1	−
RH	Star cancellation	2	−
RH	Trail Making Test A	0	0
RH	Trail Making Test B	0	0
RH	Trail Making Test B‐A	0	5
RH	Digit symbol substitution test	3	−
LH	Oral praxis	1	−
LH	Ideomotor apraxia	1	−
LH	Token test	0	1
LH	Object naming	5	−
LH	Verb naming	6	−
LH	Verbal fluency	4	10
LH	Reading words	2	−
LH	Reading pseudowords	4	−
LH	Repetition words	1	−
LH	Repetition pseudowords	4	−
LH	Phonological discrimination	0	−
LH	Auditory lexical decision	9	−
LH	Visual lexical decision	10	−

Tasks administered to the LH group showed that all the patients were normal. Visual and auditory lexical decision tasks showed a higher number of impaired patients.

**FIGURE 2:**
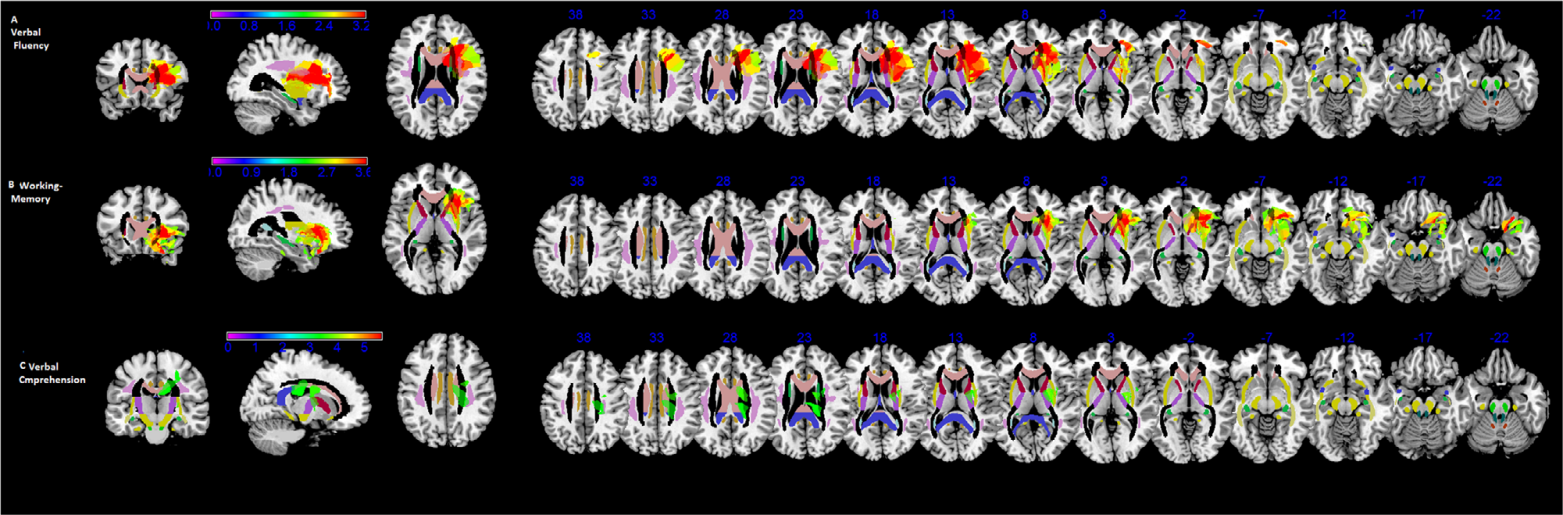
Patients’ performance expressed as equivalent scores 1–4. A score lying below or equal/above the external nonparametric tolerance limit of adjusted scores corresponds to 0 or to 4 respectively; 1 is borderline, 2 and 3 are intermediate

For tests allowing the use of equivalent scores (namely short‐term memory, working memory, verbal fluency, verbal comprehension, TMT, and constructional praxis), we used them to identify borderline performance (see Figure 2). A score lying below or equal/above the external nonparametric tolerance limit of adjusted scores corresponds to 0 or to 4 respectively; 1, 2, and 3 are intermediate (Capitani & Laiacona, 1988) (see Table 3). This suggests that for some tasks such as verbal fluency, despite patients were still within the normal range, their performance was borderline (ES = 1).

### 3.4 Correlation between epilepsy‐related information lesion‐related information and cognitive status

#### 3.4.1 LH patients

We first performed a Wilcoxon–Mann–Whitney test and found that the medians of the test scores differed significantly for the following tests and variables:

– Ideomotor apraxia test score medians significantly differed (median value: 22.92 vs. 15.43, *Z* = –2.0605, *p* = .0393) for median preoperative DT2T1 MRI >18 or <18 mm.
– Token test score medians significantly differed (median value: 23.72 vs. 12.06, *Z* = –2.4266, *p* = .0152) for median preoperative DT2T1 MRI >18 or <18 mm.
– Verbal fluency test score medians significantly differed (median value: 22.24 vs. 12.28, *Z* = –2.0306, *p* = .0211) for median preoperative DT2T1 MRI >18 or <18 mm.
– Object naming test score medians significantly differed (median value: 25.73 vs. 18.62, *Z* = 2.0212, *p* = .0433) for lesion localization in the pre‐/postcentral gyrus or in the temporal/insula area
– Verbal fluency test score medians significantly differed (median value: 14.57 vs. 23.35, *Z* = –2.2109, *p* = .0270) for EEG resulted as epileptic or normal.

We performed a bivariate Spearman's correlation analysis between the epilepsy‐related variables.

We found a correlation (inverse) between T2 lesion volume and Token test (*r* = –0.518, *p* = .0004) and working memory (*r* = –0.521, *p* = .0011) and between the DT2T1 MRI and ideomotor apraxia (*r* = –0.3869, *p* = .0113).

We then performed a multiple regression using a backward selection where we found that Token test was predicted by a model (*p* = .0078) containing the variable codifying whether the DT2T1 MRI was >18 or <18 mm, corrected for seizure features; in particular a DT2T1 MRI >18 corresponds to a reduction of 1.3 points in Token test (*p* = .0113, Parameter estimate = –1.31173).

The model containing the variable codifying the localization (pre‐/postcentral vs. temporo/insular area) and the variable codifying whether the DT2T1 MRI was >18 or <18 mm, corrected for sex, EEG, and onset significantly predicts the object naming performance (*p* = 0.0113). In particular, a lesion localization in temporo/insular area corresponds to a 1.4 point reduction in the object naming score (*p* = .0057, Parameter estimate = –1.36833) and a DT2T1 MRI >18 corresponds to a 1.3 point reduction in the in the object naming score (*p* = .0245, Parameter estimate = –1.31527).

The model containing the variable codifying the localization (pre‐/postcentral vs. temporo/insular area) and the variable codifying whether the DT2T1 MRI was >18 or <18 mm, corrected for AEDs code and EEG significantly predicts the verbal fluency score (*p* = .0056). In particular, a lesion localization in temporo/insular area corresponds to a reduction of 8.3 in the score (*p* = .0093, Parameter estimate = –13.29150) and a DT2T1 MRI >18 corresponds a reduction of 13.3 points (*p* = .0341, Parameter estimate = –8.26524).

The model containing the variable codifying the Engel class and the variable codifying whether the DT2T1 MRI was >18 or <18 mm, corrected for sex, significantly predicts the working memory score (*p* = .0117). In particular, an Engel class equal to 2, 3, and 4 corresponds to an increase in the score of 0.8 (*p* = .0313, Parameter estimate = 0.822) and a DT2T1 MRI >18 corresponds to a reduction in the score of 1.3 (*p* = .0057, Parameter estimate = –1.31981).

#### 3.4.2 RH patients

We first performed a Wilcoxon–Mann–Whitney test and found that the medians of the test scores differed significantly for the following tests and variables:

– Clock Drawing Test score medians significantly differed (median value: 13.32 vs. 3.16 *Z* = –2.7, *p* = .0069) for EEG normal or epileptic
– Clock Drawing Test score medians significantly differed (median value: 13.1 vs. 4.66 *Z* = –2.2327, *p* = .0256) for Engel class 1 or 2, 3, and 4.
– TMT A score medians significantly differed (median value: 8.83 vs. 16.84, *Z* = –2.7050, *p* = .0068) for lesion localization in the pre‐/postcentral gyrus or in the temporal/insula area.
– TMT B score medians significantly differed (median value: 8.91 vs. 16.08, *Z* = –2.4537, *p* = .0141) for lesion localization in the pre‐/postcentral gyrus or in the temporal/insula area.
– TMT B‐A score medians significantly differed (median value: 9.25 vs. 15.75, *Z* = –2.2233, *p* = .0262) for lesion localization in the pre‐/postcentral gyrus or in the temporal/insula area.

We then performed a multiple regression (backward technique).

The model containing the variable codifying Engel class, AEDs, and EEG significantly (*p* < .0001) predicts the clock drawing score. In particular an Engel class equal to 2, 3, and 4 corresponds to a reduction of 2.1 points (*p* = .0017, Parameter estimate = −2.12602), a polytherapy corresponds to an increase of 1.5 points (*p* = .0510, Parameter estimate = –1.52439), and EEG epileptic corresponds to a reduction of 3.6 points (*p* ≤ .0001, Parameter estimate = −3.63415).

The model containing the variable codifying the localization (pre‐/postcentral vs. temporo/insular area), corrected for EEG, significantly predicts TMT‐A score (*p* = .0022). In particular, a lesion localization in temporo/insular area corresponds to an increase in score of 12.9 (*p* = .0013, Parameter estimate = 12.89).

The model containing the variable codifying the localization (pre‐/postcentral vs. temporo/insular area), significantly predicts TMT B‐A score (*p* = .0373). In particular a lesion localization in temporo/insular area corresponds to an increase in the score of 28.5 (*p* = .0373, Parameter estimate = 28.500).

#### 3.4.3 Neuroanatomical results

**FIGURE 3:**
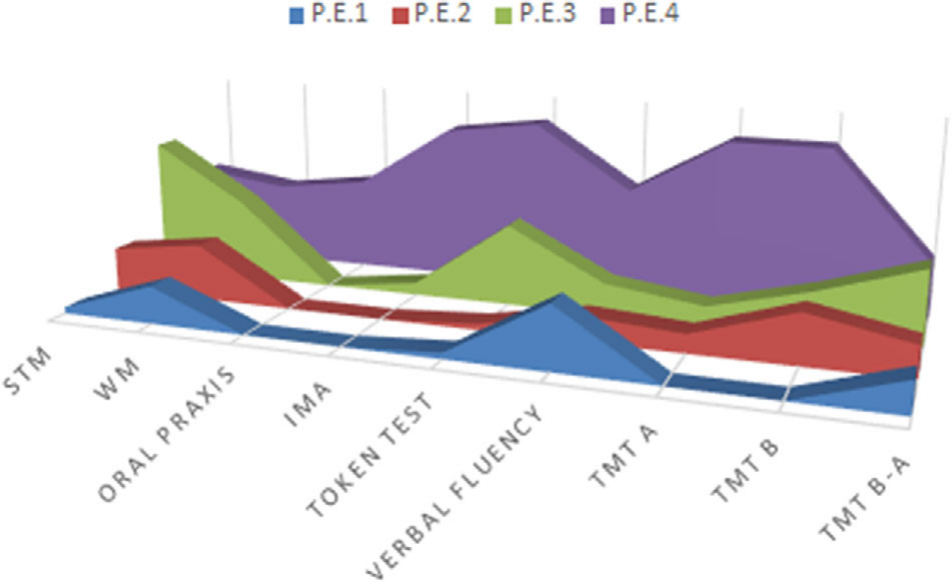
VLSM templates showing lesions significantly affecting performance on (a) verbal fluency, (b) working memory, and (c) verbal comprehension (Token test) (*p* < .01, thresholds based on permutation method). MR images are displayed following radiological convention (left is right and vice versa). Results overlap with the JUH template of white matter pathways on axial slices

Results of the VLSM analyses (see Figure 3 and Table 4) were significant for verbal fluency, verbal comprehension (Token test) and for working memory at *Z* = 3.33. We report below the significant areas corresponding to the center of mass along with the percentage of damaged voxels for each region.

**TABLE 4:** Results of the VLSM analyses

Area	Hemisphere	No. of voxels	% of total	Max Z‐score*	Max X	Max Y	Max Z
**Verbal fluency**							
Caudate	LH	3150	3,966,755	3,890,592	−20	9	15
Putamen	LH	4353	5,115,159	3,890,592	−27	12	9
Insula	LH	4845	342,936	3,890,592	−37	17	6
Superior fronto‐occipital fasciculus	LH	345	6,804,734	3,890,592	−21	3	19
Anterior limb of int. capsule	LH	1593	527,833	3,890,592	−21	7	11
Superior corona radiata	LH	2334	3,108,684	3,890,592	−23	0	19
External capsule	LH	2478	4,435,296	3,890,592	−27	12	9
Anterior corona radiata	LH	3278	4,784,005	3,890,592	−25	29	8
Inferior frontal gyrus p. operc	LH	5947	5,322,176	3,540,084	−42	15	12
Rolandic operculum	LH	3189	297,121	3,352,795	−48	−8	11
**WM**							
Insula	LH	5209	3,687,005	6,003,489	−34	18	0
Putamen	LH	5643	6,631,022	4,126,376	−27	17	−2
External capsule	LH	3132	5,605,871	4,126,376	−27	17	−2
Olfactory cortex	LH	924	4,041,995	3,890,592	−22	14	−19
Pallidum	LH	704	321,755	3,890,592	−25	−1	1
Inferior frontal gyrus p. orb	LH	4277	3,111,224	3,890,592	−20	13	−26
Anterior limb of int. capsule	LH	851	2,819,748	3,890,592	−21	19	1
Anterior corona radiata	LH	2720	3,969,644	3,890,592	−22	22	2
Caudate	LH	2859	3,600,302	3,719,017	−20	21	3
Uncinate fasciculus	LH	185	4,920,213	3,339,722	−32	1	−15
**Token**							
Superior fronto‐occipital fasciculus	LH	160	3,155,819	3,540,084	17	−11	19
Superior corona radiata	LH	2227	2,966,169	3,540,084	23	−17	19
Posterior limb of int. capsule	LH	882	2,350,746	3,890,592	22	−14	2
Putamen	LH	1274	1,497,062	3,890,592	27	−8	3
Pallidum	LH	318	1,453,382	3,890,592	24	−8	2

* Permutation, few corrections.

*Note*: The number of damaged voxels (and the % of the total no. of voxels) for each region as reported in the brain atlas of grey (AAL) and white matter (JHU and NatBrainLab) are reported, along with the center of mass (Max X, Y, and Z MNI coordinates).

The VLSM analyses performed in the LH group for verbal fluency test revealed that performance correlated with damaged voxels in the superior fronto‐occipital fasciculus (68% of this region), anterior limb of the internal capsule (52% of this region), putamen (51% of this region), anterior corona radiata (47% of this region), external capsule (44% of this region), caudate (39% of this region), insula (34% of this region), pars opercularis of the inferior frontal gyrus (53% of this region), and the rolandic operculum (29% of this region).

The VLSM analyses performed in the LH group for Token test revealed 31% voxel damaged in the superior fronto‐occipital fasciculus, 29% in the superior corona radiate, 23% in the posterior limb of the internal capsule, and 14% in the putamen and pallidum associated with a lower performance.

The VLSM analyses performed in both the LH and RH groups for working memory (verbal and visual) revealed the insula (36% of the area), the putamen (66% of the area), the external capsule (56% of the area), the olfactory area (40% of the area), the pallidum (32% of the area), the anterior corona radiate (39% of the area), the pars orbitalis of the inferior frontal gyrus (31% of the area), the caudate (36% of the area), and the uncinate fasciculus (49% of the area).

All the other tasks did not survived correction for multiple comparisons.

## 4 DISCUSSION

We studied a consecutive series of 73 patients with LGG involving the left and the right hemispheres. All patients had seizures at the time of onset and were on drug treatment with ASMs.

Our main result is that we found that performance in tasks related to language (Token test, object naming, and verbal fluency), cognitive flexibility and shifting (TMT) was predicted by lesion‐related variables. Epilepsy‐related variables predicted performance for one task only, namely visuospatial planning performance.

The little relation between ASMs (and epilepsy‐related variables) and cognitive impairment result may depend on three main aspects.

First, the majority of patients were treated with only one medication. This might limit the occurrence of cognitive adverse events and drug‐to‐drug pharmacodynamic and pharmacokinetic interactions that may result in side effects potentiation and increased drug blood levels (van Breemen et al., 2009). Nowadays, in treating LGG‐related epilepsy, a monotherapy approach is preferred. For example, in a group of 140 patients (33 LGG), (van Breemen et al., 2009) a greater use of ASM monotherapies in patients with LGG is reported (56% LGG vs. 36.7% high‐grade Glioma [HGG]). These data suggest that monotherapy approach could preserve patients from the onset of cognitive deficits that may be related to ASM.

Second, in our sample, the most represented ASM was levetiracetam (LEV), at mean dose of 1500 mg/die, which does not affect significantly cognitive functions (Lamberty et al., 2000; Zhou et al., 2008). The wide use of LEV may represent a bias in our sample; however, LEV is nowadays one of the most prescribed ASMs for TRE. LEV and other newer ASMs (i.e., lacosamide, perampanel, and brand new brivaracetam) are reported by guidelines and expert opinions as the best choice to treat TRE, for their favorable pharmacokinetic profile and lack/limited cognitive side effects (Maschio et al., 2019). By contrast, old generation ASMs, especially enzyme‐inducing ASMs, have side effects exerting also on cognitive functioning and on patients’ mood as they may generate depression and irritability (Maschio, 2012; Maschio et al., 2019).

Lastly, in our sample, mean duration of epilepsy was 5.8 months, thus the role of recurrent seizures in generating possible cognitive impairment appears less important comparing with long‐term epilepsies. A recent study (Gavrilovic et al., 2019) demonstrated a higher degree of cognitive impairment measured with Montreal Cognitive Assessment (MoCA) in patients with drug resistant epilepsy.

Taken together, our results add further information on the effect of TRE on cognition and complement previous literature, which may appear inconsistent, showing that this issue deserves further studies. For example, mood cognition and quality of life were examined in a study (Rahman et al., 2015) involving a cohort of 81 patients affected by gliomas (both LGG and HGG), medulloblastomas and meningiomas with 55 of them presenting also TRE. It was reported that cognitive impairments occurred in 50.6% of their subjects, but this happened regardless the presence of epilepsy and the use of ASMs. It is worthy to mention that, unlike our population, many patients were under polytherapy and were taking carbamazepine and valproate. It is also worthy to mention that cognitive status was measured using the MoCA and frontal assessment battery tests. We complement these results, by adding extended information about the patients’ neuropsychological status.

In another study (Maschio et al., 2014), investigating the burden of epilepsy in patients with brain tumors, quality of life was significantly affected by presence, type, and duration of therapy with ASMs. In particular, patients perceived a negative effect on cognition and social function. Interestingly, this occurred independently of the ASM used. Moreover, this subjective perception was stronger in patients receiving ASMs for periods longer than 6 months. These data came all from subjective scale evaluating quality of life, which were collected at the first neurological visit. No statistically significant difference has been observed regarding Mini Mental State Examination scores. Thus, neither the tumor nor epilepsy and its treatments influenced the neurological functions of the subjects examined and ASMs seemed to affect patients’ cognitive status more from a subjective point of view (Khan & Anatya, 2013; Maschio et al., 2014). In our sample, short duration of epilepsy, prevalent antiseizure monotherapy, and first diagnosis of LGGs, with all the expectations related to surgery, which represents, in most of cases, the prominent therapeutic act, may contribute to reduce the perception of illness burden.

In a further study (Derks et al., 2019), authors compared patients with IDH wild‐type LGG and patients with IDH mutated LGG and found that the former presented a poorer cognitive status. This result was obtained regardless age, presence of epilepsy, and concomitant use of ASMs. Patients with IDH wild‐type gliomas showed a lower functional connectivity in the alpha band, evaluated with magnetoencephalography. Increased alpha band connectivity has been demonstrated to correspond to improve cognitive functions (van Dellen et al., 2012). Authors (Derks et al., 2019) speculate that the overexpression of D‐2‐hydroxyglutarate due to IDH mutation may contribute to explain the difference in alpha band connectivity. In fact, D‐2‐hydroxyglutarate mimics the effect of glutamate binding to NMDA and AMPA receptors; glutamate is the main excitatory neurotransmitter, thus enhancing both the occurrence of epileptic seizures and neuronal synchronization. This work emphasized tumor characteristics as mainly involved in determining cognitive status of subjects affected by gliomas. In this way, it is in line with our results and it may explain why the presurgical cognitive status was within normal range in our sample: in fact, the majority of patients had IDH‐mutated gliomas.

### 4.1 Neuropsychological results

From a neuropsychological point of view, patients perform within the normal range in most tests.

The normal cognitive performance of our patients supports the idea that a monotherapy treatment is preferable to a polytherapy. This avoids the toxicological effect of the use of several drugs at the same time (Klein et al., 2003). Not only new ASMs cause fewer side effects on cognitive functioning than old ASMs (Klein et al., 2003). This is also confirmed in our work where patients’ cognitive performance is mostly within the normal range. The most of our sample (90.5%) have a monotherapy drug treatment. 54/73 (73.9%) patients assumed LEV. As reported in literature (Vecht & Wilms, 2010), LEV has a good tolerability, reduces 50% or more seizures in 2/3 of patients, and has significant fewer cognitive side effects. LEV is a second‐line ASM and presents higher efficacy in patients with seizure (van Breemen et al., 2009).

### 4.2 Neuroanatomical results

In our LH‐group VLSM maps revealed an involvement of the precentral gyrus, frontal (superior and middle) gyrus, inferior frontal, insula, anterior cingulate, caudate, putamen/globus pallidus, and left‐thalamus in verbal fluency. This is a classical neuropsychological test of language production where subjects have to generate and articulate words in response to a phonological cue. Neurocognitive models define an important role of the left frontal executive regions in phonological fluency (Mummery et al., 1996). Results are similar to other studies about verbal fluency, showing a role of the left insula, putamen, superior temporal pole, and external capsule (Pisoni et al., 2019), and the left putamen, caudate nucleus and pallidum, and temporal region (Chouiter et al., 2016).

In our VLSM analyses, we found an involvement of the insula, putamen, and frontal structures, consistently with previous functional neuroimaging literature (Ivanova et al., 2018; Wager & Smith, 2003). Other authors (Grahn et al., 2009) and (Ivanova et al., 2018) have found activation in WM task also in caudate, as we did in our sample in addition to frontal areas.

As far as the Token test is concerned, in a VLSM study (Pisoni et al., 2019) authors found task impairments correlated with damaged voxel in posterior part of the left superior and middle temporal gyri as well as with the angular and supramarginal gyri. Other authors (Papagno & Cecchetto, 2019) argued that as the Token test involves short‐term memory component, significant voxels are found in temporo‐parietal areas (Pisoni et al., 2019). Authors identified voxel damage correlating to impaired performance at the Token test in the middle and superior temporal gyrus, the supramarginal gyrus, angular, and in subcortical areas. The large network involved in language comprehension includes also subcortical structures such as basal ganglia, especially the caudate and the thalamus, as we found in our VLSM analysis.

## COMPETING INTEREST

None of the authors has any conflict of interest to disclose.

## AUTHOR CONTRIBUTIONS

G.P. T.I., and B.T. designed the research; I.G., G.P., T.I., and M.S. performed the research; F.P. analyzed the data; I.G., B.T., and G.P. wrote the paper; all authors edited the paper; I.G., B.T., G.P., T.I., and M.S. collected the data; all authors revised the final version of the manuscript.

### PEER REVIEW

The peer review history for this article is available at https://publons.com/publon/10.1002/brb3.2560.

## Supporting information

SUPPORTING INFORMATION

## Data Availability

Data will be shared upon request by contacting the corresponding author.
