# Supplementary material for: Presurgical cognitive status in patients with low‐grade glioma and epilepsy: Testing the effects of seizures, antiseizure medications, and tumor localization
Source: Brain Behav. 2022 Apr 4;12(5):e2560. doi: 10.1002/brb3.2560 (PMC9120733; doi:10.1002/brb3.2560)
Supplement: Supplementary file 1 — SUPPORTING INFORMATION [file BRB3-12-e2560-s001.doc]

**Supplementary Table 1**

**Spatial coordinates, according to the Montreal Neurological Institute space system, of the brain areas listed in the first column, with the worst damage, as evidenced by the percentage lesion overlay indicating the percentage of patients having a lesion maximally localized in the listed area.**

| **Area** | **Hemisphere** |  | **x** | **y** | **z** | **% overlap** |
| --- | --- | --- | --- | --- | --- | --- |
| Insula | LH | -37 | 5 | -9 | 22 | 52,38095 |
| External capsule | | -34 | -4 | -8 | 21 | 50 |
| Putamen | LH | -34 | -6 | -7 | 21 | 50 |
| Uncinate fasciculus | | -33 | 2 | -19 | 20 | 47,61905 |
| Amygdala | LH | -33 | 2 | -19 | 20 | 47,61905 |
| STG | LH | -44 | 2 | -15 | 20 | 47,61905 |
| Temporal pole | LH | -34 | 5 | -23 | 20 | 47,61905 |
| Pallidum | LH | -27 | -3 | -2 | 19 | 45,2381 |
| Hippocampus | LH | -38 | -8 | -18 | 18 | 42,85714 |
| IFG | LH | -34 | 15 | -19 | 18 | 42,85714 |
| Parahippocampal gyrus | LH | -28 | 6 | -25 | 17 | 40,47619 |
| Sagittal stratum (IFOF+ILF) | | -37 | -9 | -18 | 17 | 40,47619 |
| Precentral gyrus | RH | 39 | 5 | 19 | 14 | 45,16129 |
| Rolandic operculum | RH | 40 | 4 | 14 | 14 | 45,16129 |
| Insula | RH | 45 | 3 | 9 | 13 | 41,93548 |
| Superior corona radiata | RH | 32 | -4 | 22 | 13 | 41,93548 |
| Superior longitudinal fasciculus | RH | 35 | -1 | 19 | 13 | 41,93548 |
| Heschl's gyrus | RH | 37 | -29 | 12 | 12 | 38,70968 |
| External capsule | RH | 29 | 11 | 14 | 12 | 38,70968 |
| IFG | RH | 44 | 17 | 5 | 11 | 35,48387 |
